# The importance of colostrum in maternal care and its formation in mammalian species

**DOI:** 10.1093/af/vfad012

**Published:** 2023-06-14

**Authors:** Craig R Baumrucker, Josef J Gross, Rupert M Bruckmaier

**Affiliations:** Department of Animal Science, Penn State University, University Park, PA 16802; Veterinary Physiology, Vetsuisse Faculty, University of Bern, 3012 Bern, Switzerland; Veterinary Physiology, Vetsuisse Faculty, University of Bern, 3012 Bern, Switzerland; Veterinary Physiology, Vetsuisse Faculty, University of Bern, 3012 Bern, Switzerland

**Keywords:** colostrum, FcRn, lactoferrin, mammary, regulation

**Implications**

- Colostrum formation is directed by placental structure and hormone signals
- Quality colostrum is defined by the concentration of immunoglobulin
- Quality colostrum formation in ungulates (hoofed mammals) is dependent on the Fc receptor of the neonate (FcRn)
- While the FcRn binds IgG1, IgG2, and albumin, only IgG1 is transported to colostrum
- FcRn IgG release into colostrum is pH dependent and lactoferrin likely facilitates it release

## Introduction

Members of the class mammalia are so named for their mammary glands. For the newborn of mammalia, the first secretions of the mammary gland are either helpful or critical for early development and health. Great variation exists among species with respect to the course of mammary development and the factors regulating colostrum (first milk) formation. Following birth, all mammalian neonates suckle on the colostrum whose components include many hundreds to thousands of distinct bioactive molecules that protect against infection and contribute to immune maturation, intestinal development, and healthy microbial colonization (Ballard and Marrow, 2013). Most critical for all early neonatal health are the immunoglobulins, also known as antibodies, which are either provided to the fetus in utero or to the newborn early after birth. They act as a critical part of the immune response by specifically recognizing and binding to particular antigens, such as bacteria or viruses, and aid in their destruction. The antibody immune response is highly complex and very specific, but simplistically, antibodies attach to a specific foreign protein and make it easier for the immune cells to destroy that protein. The various immunoglobulin classes (IgA, IgD, IgE, IgG, and IgM) and their subclasses (isotypes within each class) differ in their biological features, structure, target specificity, and distribution, especially those in the colostrum.

Colostrum is very different than mature milk and individual species have different strategies for the onset and termination of the colostrum-producing period. One example of this difference is IgA antibodies occur in almost all of the external secretions of mammalia—with the exception of the ungulate (mammals with hooves: i.e., cattle, pigs, camels, sheep, deer, and many more) mammary gland that mainly secretes IgG. Another example of this variation is that ungulate species form colostrum with IgG in a period of 3–4 weeks before birth ending shortly after partum, while the rodent species continue colostrum production over the entire suckling period (21 d) with an IgA emphasis that gradually transitions to mature milk. In addition to the emphasis on different classes of immunoglobulins, the provision of different subclasses of immunoglobulins also occurs in colostrum. There are four subclasses of IgG (IgG1–4) and the different subclasses are more than 90% similar. Nevertheless, their effector functions and affinity for antigens can vary significantly. IgG1 and IgG2 are the most abundant subclass in ungulate blood serum, but only IgG1 is selectively transported to colostrum because of its unique structure. All these differences are related to placental and mammary immunoglobulin transport mechanisms (Butler and Kehrli, 2005; Baumrucker et al., 2021).

### The placenta resolves: the co-evolution of gestation and early lactation

One of the most important tasks of maternal care in mammalian species is the transfer of components of the adaptive immune system from the mother to the offspring. This is a passive immunization for the early neonatal life to bridge the time until the newborn produces its own immunoglobulins and other immuno-active components. Thus, if provided, the life of the newborn starts with the full arsenal of maternal immunoglobulins specifically directed against pathogens in the respective environment. The placental structure dictates the capability of the placenta to transport immunoglobulins from maternal to fetal circulation. Depending on the placenta type, and the number of tissue layers between the maternal and fetal blood, passive immunization may or may not occur in utero. With an increasing number of layers the capacity to transport immunoglobulins in utero is reduced until immunoglobulin transport does not occur. In the latter case, the newborn offspring depends on the oral intake of immunoglobulins and other bioactive components via the first mammary colostrum secretions. A typical species with intra-uterine transfer is human (Butler, 1974; Bigler et al., 2022) while ungulates constitute the other extreme.

In the ungulate species where IgG1 protein cannot pass the placental barrier in utero, passive immunization occurs by colostrum consumption following birth. In these species, the immediate availability of colostrum with a high concentration of immunoglobulins allows the intestinal uptake of intact immunoglobulins. However, maximal ungulate neonate intestinal absorption occurs within the first hours following birth, and when induced by colostrum, absorption decreases with time to where the gut is closed to intact uptake (24 h). Therefore, sufficient colostrum must be available immediately after birth and the newborn must simultaneously be ready for feeding. Ungulate species give birth to offspring which are able to find the mammary gland and start to suckle shortly after birth.

The formation of pre-colostrum is the active transfer of immunoglobulin and other bioactive components into mammary secretions. It starts days or weeks before birth in the cattle species (Baumrucker and Bruckmaier, 2014). This process has been termed colostrogenesis. For successful suckling of the newborn, colostrum must be available in a sufficiently liquid form, with adequate immunoglobulins, nutritive components, and a variety of non-nutritive components such as hormones and growth factors. The latter have been shown to affect gastrointestinal development (Blum, 2006). While the rather thick pre-colostrum is forming, timely initiation of lactogenesis (beginning of mature milk) during the latter prepartum period is a prerequisite for an adequate watery colostrum composition to facilitate neonatal intake. Lactogenesis provides nutritive components such as carbohydrates (lactose), fat, and proteins that are synthesized in the mammary cells. It is lactose that mainly determines the amount of water in colostrum and milk through the progress of an osmotic gradient between the cytoplasm and secretory vesicles of the mammary cells.

### Endocrine mechanisms to start colostrum and milk secretion

The immediate availability of colostrum after birth is either crucial or desirable for the optimal health of the newborn. In species with placental passive immunization, such as humans, colostrum is not as essential for systemic immunization. Also, in these species, immunoglobulins of the IgA class are lower in concentration in the colostrum and the mammary transport system is different than that of IgG-transporting species. IgA has a different transport system than that of IgG1 and IgA presence has been shown to be effective in the neutralization of bacterial toxins at the intestinal mucosal surface of the newborn (Neville, 1999).

During pregnancy, different steroids are produced by the uterus and conceptus and as pregnancy progresses, estrogen and gestagens (progesterone and analogs) concentrations increase to induce the growth and development of the mammary gland (Tucker, 2000). These circulating hormones are known to stimulate local mammary factors and receptors in special cells that direct mammary progression. Progesterone (P4) exerts the strongest activity in most species. Its high concentration during the final weeks of pregnancy appears to induce the events that initiate colostrogenesis. Progesterone declines around birth and subsequent expulsion of the conceptus permits the beginning of secretion of mature milk constituents. This decline in P4 also allows the binding of cortisol to bind to its receptor, and with increases in prolactin secretion from the pituitary, copious milk secretion is activated.

While most species produce P4 in the ovary, in some species P4 production is superseded by the placenta during the course of pregnancy. This production has consequences for the cessation of P4 production at the end of pregnancy. In cattle species, the decrease of P4 occurs 1–2 days before birth and is due to the loss of a temporary structure located in the ovary (corpus luteum) that produces P4 and other hormones. Thus, milk secretion and hence dilution of the pre-colostrum secretion starts at this time and provides liquid colostrum at first milking (Gross et al., 2014). In contrast, P4 decreases only after placental expulsion in humans which is the reason for extended colostrum production and delayed commencement of milk secretion for up to 4 days (Neville, 2001).

Fleet et al. (1975) suggested that the transfer of IgG into the mammary gland before birth seems to be independent of the processes related to the start of milk secretion. The endocrine signals both to initiate and to cease the transfer of immunoglobulin (colostrogenesis) to form specific colostrum constituents are still unclear. Observational results from cows showed that the transfer of IgG1 into mammary secretions continues until birth (Gross et al., 2014). While most ungulate species initiate milk secretion by the drop in P4, exceptions are noted. Sheep and horses are different where the gestagen production at the end of pregnancy takes place in the placenta. These ungulate species have developed different strategies to allow early initiation of lactogenesis before placental expulsion. In sheep, the placental steroid synthesis from the precursor pregnenolone changes from P4 to estrogens which causes a decline of P4 and a simultaneous increase of placental estrogens (Liggins et al., 1977). In horses, P4 is further metabolized to gestagen metabolites which present a lower gestagen activity than P4 (Legacki et al., 2018). In species where the corpus luteum (temporary endocrine structure in the ovary that is involved in the production of P4 and estradiol) produces the P4 throughout pregnancy, the production of prostaglandin F2α occurring from the uterus or ovary induces a reduction in P4 that occurs prior to birth. This is the case for the cow, goat, pig, dog, cat, rabbit, and rodents and is considered to be the key to initiate both partum, colostrogenesis termination, and copious mature milk production.

The signal for P4/gestagen withdrawal, whether placental or luteal, originates in the fetus in most species. The maturation of the fetus and activity of the hypothalamus–pituitary–adrenal axis leads to an increased release of cortisol from the fetal adrenal cortex, which, through species-specific enzyme activation, triggers the endocrine changes related to both birth and lactogenesis (Schuler et al, 2018).

### Focus on cattle colostrum composition

Cow colostrum is typically rather thick with an average density of 1.048 g/mL. It is yellowish due to dietary carotenoids that are soluble in milk fat globules and mildly acidic (pH 6.0) when compared to mature milk (pH 6.8). Immunoglobulins make up 70–80% of the total protein in colostrum (Butler and Kehri, 2005), which accounts for the thickness when immunoglobulin concentration is high. Immunoglobulins present in cow colostrum include G, M, and A, but it is IgG subclass 1 (IgG1) that constitutes 65–80% of immunoglobulin in cow colostrum (Table 1). Of the four IgG subtypes IgG1 and IgG2 predominate in cattle maternal blood and concentrations are nearly equal at around 10 mg/mL before colostrum formation. Remarkably, only IgG1 is selectively transported to high concentrations in colostrum (Table 1 ratio milk/blood). During the colostrum forming period, blood IgG1 declines to low levels (~1 mg/mL), presumably to provide the immunoglobulin to colostrum. However, the IgG1 concentration in cow colostrum is known to be extremely variable (10 mg/mL to >166 mg/mL; Baumrucker et al., 2010). Because of the neonatal need for a high mass of immunoglobulins in a concentrated liquid immediately following birth, the so-called “quality” of colostrum is dictated by IgG1 concentration. Research has shown that calf consumption of igG1 concentration below 50 mg/mL increases the risk of morbidity and mortality of the calf.

**Table 1.** Immunoglobulins (IgX), lactoferrin (Lf), and serum albumin (BSA) concentration, mass, and ratio in colostrum when compared to that of blood serum

Factor	Mean ± SEM Colostrum	Min-max Colostrum	Mean ± SEM Colostrum	Min-max Colostrum	Mean ± SEM Blood	Ratio mg/mL Colostrum/Blood
Measure	mg/mL	mg/mL	Grams	Grams	mg/mL	Mean vs. Max
BSA	1.2 ± 0.5	0.4–2.6	2.2 ± 1.8	0.1–9.7	36.5 ± 5	0.03–0.07
IgG1	34.96 ± 12.2	11.8–74.2	291.6 ± 315	14–2,223	1 ± 0.3 ^a^	35–74
IgG2	6.00 ± 2.8	2.7–20.6	3.9 ± 2.3	0.2–10.3	13.3± 0.4	0.45–1.5
IgA	1.66 ± 1	0.5–4.4			0.41	4.0
IgM	4.32 ± 2.8	1.1–21.0			2.4	1.8
Lf^*^	45 ± 135	0.25–267	195.9 ± 453	11–31,558	0.2	

Data from Baumrucker et al., 2021;

^*^unpublished data (Baumrucker & Macrina, 2023).

^a^ Blood concentration at week before birth

Cow serum albumin (BSA) has been included in Table 1 because it has been shown to bind to the receptor that has been shown to transport IgG1 to colostrum. However, it is not concentrated in colostrum. In addition, we have added lactoferrin to Table 1 because it has a strong positive relationship with IgG1 concentration and mass in the first-milked colostrum (Baumrucker et al, 2021). Lactoferrin is synthesized in mammary cells and secreted into colostrum and therefore a milk/blood ratio is not listed (Table 1). Of the hundreds to thousands of distinct bioactive molecules (Grosvenor et al., 1993) that exist in colostrum, only some of these are expressed at concentrations that exceed that of blood, but not in milk. These factors in high concentration are shown in Table 2 with high milk/blood ratios. These belong to the insulin-like growth factor family of proteins that are known for their cellular growth, developmental, and survival mechanisms (LeRoith et al., 2021). They appear to be integrated into the IgG1 secretion mechanism described below.

**Table 2.** Growth factors of the Insulin-like growth factor family that are high in colostrum. Data are concentrations in bovine colostrum, milk, blood, and ratios

Factor	Colostrum	Milk	Blood	Max ratio C/B
IGF-1	312–1,850 ng/mL	2–10 ng/mL	20–225 ng/mL	92.5
IGF-2	394–1,500 ng/mL	1–20 ng/mL	150–173 ng/mL	10
IGFBP-3	2–3X	1X	1X	2–3
Insulin	53–106 ng/mL	4–10 ng/mL	0.2–1.2 ng/mL	>100

Data from Baumrucker and Macrina, 2022. C/B is colostrum/blood concentration.

X is relative concentration.

### Quality assessment of colostrum from dairy cows, sheep, and goats

In order to meet the needs of the neonate, milking of colostrum should occur as early as possible after birth. With the closure of the blood-milk barrier and the onset of copious milk production after birth, the quality of colostrum declines rapidly with time. Due to the significant variation of IgG1 concentration, the control of colostrum quality is essential for successful offspring rearing. Colostrum quality refers particularly to the content of immunoglobulins. Laboratory methods like radial immunodiffusion assays or enzyme-linked immunosorbent assay that are directly measuring immunoglobulins are rated the “gold standards” in terms of precision and accuracy. However, these methods are time-consuming, expensive, and not suitable in farm practice. On-farm instruments for the immediate testing of colostrum (e.g., colostrometer, Brix refractometer) only indirectly assess colostral immunoglobulin content. Colostrum quality estimations of the latter methods depend largely on physical and chemical properties like density, viscosity, color, and the contents of fat and protein. Specificity and sensitivity are therefore much lower compared with the gold standard methods. However, the simple handling and immediate availability of results give a fairly good estimate to distinguish between poor and superior colostrum quality, although conclusions about the immunoglobulin content need to be interpreted with caution.

However, colostrum yield and constituents do not only differ between animals and farms but also vary considerably among individual mammary gland quarters in cows (Samarütel et.al., 2016). Although not practicable, the collection of colostrum at an individual mammary quarter (or udder half, resp.) level instead of milking and merging all colostrum could theoretically provide the best fraction for the offspring. A study by Vetter et al. (2013) showed that colostral immunoglobulin contents remain constant throughout the milking of the entire udder. A potential negative relationship between colostrum yield and IgG content is currently not assumed (Gulliksen et al., 2008; Kessler et al., 2014, 2020a). In dairy cows, genetic selection for higher milk production did not result in a poorer colostrum quality when compared with less intensively selected cows (Kessler et al., 2020b). However, meat-oriented sheep and goat breeds, with less colostrum volume, seem to have greater IgG concentrations in colostrum compared to dairy breeds (Kessler et al., 2019). Despite similarities in colostrum formation, further species differences in colostrum composition (i.e., contents of fat, protein, and lactose) exist between sheep, goats, and cattle. Interestingly, both colostrum yield and IgG content were observed to be highly repeatable, even at a quarter level, in consecutive lactations of dairy cows (Gross et al., 2016).

### Fc receptor of the neonate: FcRn

Because the newborn calf does not receive immunoglobulins in utero, the provision of good-quality colostrum is critical. The concentration of cow colostrum is variable due to a number of factors, including breed, parity, dry period, and time of postpartum collection (Godden et al, 2019). With the exception of collection time and its colostrum dilution effect, only individual cow differences can explain the extreme colostrum quality issue.

In the 1960s, the existence of an intestinal receptor responsible for the efficient intestinal cellular transfer of IgG from mother colostrum to the young was first proposed by Brambell (1966). The intestinal receptor is now known as the Fc receptor of the neonate (FcRn) and binds the three most abundant serum proteins: IgG1, IgG2, and BSA in all species studied to date. Furthermore, the binding and release of these three proteins are strictly pH dependent (bind <pH 6.5; release >pH 6.5). The low cellular pH is achieved after cell inclusion by the action of the V-ATPase proton pump (H+) allowing these internalized proteins at a low pH to bind to the FcRn.

While the proteins are FcRn bound in the cell they are protected from extracellular and cellular degradation. Subsequently, they are recycled to the extracellular space. This is the explanation for the extremely long serum half-lives of >20 days for these proteins. The magnitude of FcRn rescue of these proteins has been demonstrated with animals deficient in FcRn where serum levels of IgG and BSA are 70–80% lower in the circulation with greatly shortened half-lives (Pyzik et al., 2019).

It has been shown that the cow FcRn binds all three proteins. During the colostrogenesis phase of mammary development, there is a selective transfer, termed transcytosis, but only IgG1 is transfered to the forming colostrum while IgG2 and BSA are recycled. Recently, Baumrucker et al. (2021) provided a hypothesis that the FcRn transcytosis of IgG1 and recycling of IgG2 occurs with very slight, but significant differences in the amino acid structure of the two immunoglobulins.

The main reason for the IgG1 variation in first-milked colostrum among cows of the same breed and with similar nutrition must be a defective transcytosis system. The FcRn shares a broad tissue distribution in mammals and is highly expressed in mammary epithelial cells (Kacskovics, 2004). One defect in this system could be that transcytosis is slow and the length of the dry period is inadequate to accumulate the IgG concentration. However, experiments have shown that transcytosis of IgG1 can be very fast in some animals (Baumrucker et al., 2016) and slow transcytosis could be considered a defect. A surprise finding was that with prepartum milking and removal of the forming colostrum, the IgG1 mass collected after birth represented seven times the mass that was present in the individual cow blood. The additional source of IgG1 is currently unknown. Furthermore, if cows are milked continuously without a dry period during the latter part of pregnancy, colostrum formation still occurs. However, it is mixed with copious mature milk secretion during the last week prepartum. We explain some of the possible mechanisms of transcytosis below.

### Colostrum secretion paths

**Figure 1.**
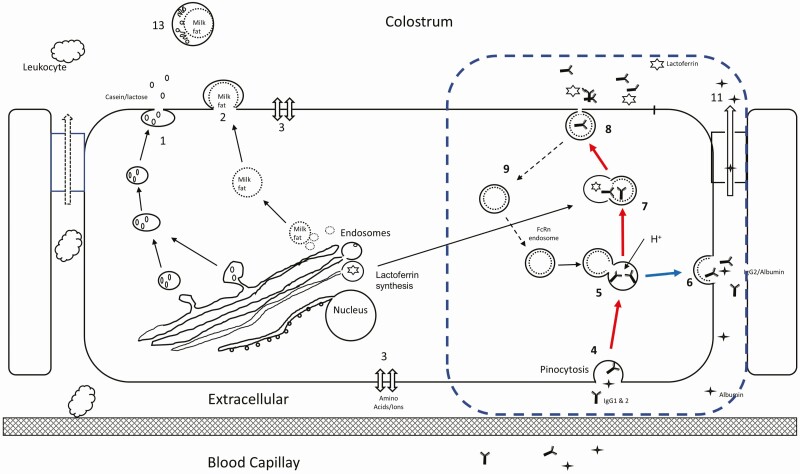
Mechanisms of colostrum production. Pathways 1 and 2 are dominant during copious milk secretion accounting for casein, lactose, and fat. The separate pathway for colostrum formation is shown in the dashed blue rectangle. Pathway 4,5,7, & 8 (red arrows) provides a separate path for extracellular IgG to enter the cell and associates with FcRn (5) and the V-ATPase lowers the pH (H^+^) to provide binding. The FcRn:IgG2 bound components are directed to be recycled (path 6, blue arrow) while FcRn:IgG1 components are directed toward colostrum (red arrows). The addition of lactoferrin (7) with the FcRn:IgG1 complex enables the release of the IgG1 and subsequent secretion into the forming colostrum (8) with the FcRn endosome recycled (9).

Mammary cells have multiple mechanisms of secretion during colostrogenesis and lactation and some of these are shown in Figure 1. In the first-milked colostrum, IgG1 is mainly located in the colostrum whey (milk serum without fat and casein). However, the FcRn protein is not found in milk or colostrum (Reinhardt and Lippolis, 2006, 2008). This finding of no FcRn in colostrum indicates that the FcRn does use the secretion pathway of casein and other soluble mammary cell components as shown in Figure 1: Pathway 1. This is also supported by the knowledge that the FcRn requires low pH for ligand binding and such an environment could not be shared with secretory components because caseins would be precipitated under such low pH conditions and the colostrum would not be fluid. Therefore, the FcRn secretion pathway, shown with a dashed blue rectangle (Figure 1: Pathway 4–9, red arrows), must be a separate and independent path than that of the casein/lactose pathway of mature milk secretion. But how does the FcRn release the binding of the IgG1 protein when forming colostrum has a pH of 6.0. We have proposed the IgG1 release occurs in the presence of high concentrations of lactoferrin (Schanbacher et al., 1993). The mammary cell synthesizes and secretes a high mass of lactoferrin during the non-lactating period when milking has stopped (Wang et al., 2019) and the latter colostrum-forming period. Lactoferrin concentration is also highly variable in colostrum with concentrations that are 30- to 100-fold higher than that of milk (Indyk et al. 2006). Lactoferrin belongs to the basic proteins with a pKa of 8.7 and we hypothesized that it provides the means to facilitate the intracellular pH increase to facilitate the release of IgG1 from the FcRn. In support of this proposal is that IgG1 and lactoferrin have a strong mass relationship (Baumrucker et al., 2021) and suggests the employment of the same secretion pathway (Figure 1, Pathway 7–8) with protein from extracellular and intracellular locations. A recent analysis of lactoferrin in colostrum (Table 1) shows great variation in concentration and mass. We have hypothesized that low lactoferrin availability in this pathway would not allow for the release of IgG1 into colostrum and provides another explanation of extreme colostrum quality. Finally, because the components of Table 2 appear in high concentration in colostrum, but not in milk, we have also proposed that these bioactive components are merged or contained in the FcRn pathway, but not bound to the FcRn complex. The mechanisms of their inclusion into the FcRn pathway are currently unknown.

## Conclusion

The species variation in the formation and composition of colostrum serves the individual neonate. However, what explains the great compositional variation within a species is little understood and remains a problem in all mammalian neonatal health. Much new research is needed to identify these mechanisms and pathways so that the variation in colostrum composition may be understood and reduced to provide a high-quality colostrum that is available to the neonate for a healthy and productive life.

## About the Authors

**Dr. Craig R. Baumrucker** (crb@psu.edu) has engaged in 48 years of scholarly activity in the area of mammary gland biology. He remains as a professor emeritus at The Pennsylvania State University (PSU), University Park, PA, USA. He received his PhD from Purdue University, West Lafayette, IN USA in 1974. He has continued his research activities with yearly visits with the University of Bern, Switzerland. His research approach is basic to define and understand mechanisms of action in mammary cells. His expertise is cell biology and endocrinology that encompasses biological membrane transporters, endocrine regulation of growth and differentiation of mammary cells, and endocrine and growth factors in colostrum. Most recently his efforts have concentrated on the role and mechanism of the Fc Receptor of the Neonate (FcRn) in colostrogenesis. His recent reports in this area have provided new insights into colostrogenesis and provide opportunities for improvement of colostrum quality and better neonatal health.

**Josef Gross** received his Ph.D. from the Technical University of Munich in animal nutrition and physiology in 2011. He is currently lecturer and senior scientist at the Veterinary Physiology group at the University of Bern, Switzerland. With more than 80 peer-reviewed publications, Dr. Gross is an internationally recognized scientist. His research activities address transition dairy cow health and metabolism, colostrum quality, the impact of nutrients and metabolites on adaptation physiology, and metabolic robustness of high-yielding dairy cows.

**E-mail:** josef.gross@unibe.ch

**Dr. Rupert M. Bruckmaier** is the head of the Veterinary Physiology of the Vetsuisse Faculty, University of Bern, Switzerland. He has been working on the physiology and endocrinology of lactating ruminants. His work contributed to the knowledge in the field of physiology and biotechnology of milk removal in different species, the metabolism of dairy cows, and the immune response of the mammary gland. Within his interest in mammary gland biology, colostrum secretion and composition has been an important topic to investigate the various immunological, endocrine and further bioactive components in colostrum. During all research activities he has tried his best to support young scientists for their future career. E-mail: rupert.bruckmaier@unibe.ch

**E-mail:** rupert.bruckmaier@unibe.ch
